# Study protocol: münster tinnitus randomized controlled clinical trial-2013 based on tailor-made notched music training (TMNMT)

**DOI:** 10.1186/1471-2377-14-40

**Published:** 2014-03-02

**Authors:** Christo Pantev, Claudia Rudack, Alwina Stein, Robert Wunderlich, Alva Engell, Pia Lau, Andreas Wollbrink, Alex Shaykevich

**Affiliations:** 1 Institute for Biomagnetism and Biosignalanalysis, University of Münster, Malmedyweg 15, 48149 Münster, Germany; 2 ENT Department, University Clinic Münster, University of Münster, Cardinal-von- Galen Ring 10, 48149 Münster, Germany; 3 2/22 Foyle Road, Bayswater, WA 6053, Australia

**Keywords:** Tinnitus, Tailor-made notched music training (TMNMT), Münster Tinnitus randomized controlled Clinical Trial-2013

## Abstract

**Background:**

Tinnitus is a result of hyper-activity/hyper-synchrony of auditory neurons coding the tinnitus frequency, which has developed to synchronous mass activity owing the lack of inhibition. We assume that removal of exactly these frequency components from an auditory stimulus will cause the brain to reorganize around tonotopic regions coding the tinnitus frequency. Based on this assumption a novel treatment for tonal tinnitus - tailor-made notched music training (TMNMT) (Proc Natl Acad Sci USA 107:1207–1210, 2010; Ann N Y Acad Sci 1252:253–258, 2012; Frontiers Syst Neurosci 6:50, 2012) has been introduced and will be tested in this clinical trial on a large number of tinnitus patients.

**Methods and design:**

A randomized controlled trial (RCT) in parallel group design will be performed in a double-blinded manner. The choice of the intervention we are going to apply is based on two “proof of concept” studies in humans (Proc Natl Acad Sci USA 107:1207–1210, 2010; Ann N Y Acad Sci 1252:253–258, 2012; Frontiers Syst Neurosci 6:50, 2012; PloS One 6(9):e24685, 2011) and on a recent animal study (Front Syst Neurosci 7:21, 2013).

The RCT includes 100 participants with chronic, tonal tinnitus who listened to tailor-made notched music (TMNM) for two hours a day for three months. The effect of TMNMT is assessed by the tinnitus handicap questionnaire and visual analogue scales (VAS) measuring perceived tinnitus loudness, distress and handicap.

**Discussion:**

This is the first randomized controlled trial applying TMNMT on a larger number of patients with tonal tinnitus. Our data will verify more securely and reliably the effectiveness of this kind of completely non-invasive and low-cost treatment approach on tonal tinnitus.

**Trial registration:**

Current Controlled Trials ISRCTN04840953

## Background

Over the past 15 years, we have studied the plasticity of the human auditory cortex by means of magnetoencephalography (MEG). Two main topics nurtured our curiosity: the effects of musical training and the effects of lateral inhibition. Our studies demonstrated that short-term and long-term musical training was able to induce functional plasticity changes in the human auditory cortex. Listening to notched music for three hours inhibited the neuronal activity in the auditory cortex that corresponded to the center frequency of the notch [1]. In a previous MEG study we have investigated the masking effect on a certain frequency region of the human auditory cortex [2] as based on quasi-simultaneous performance of habituation and lateral inhibition. Crucially, the overall masking effect on human auditory cortical activity was stronger in case of lateral inhibition than in case of habituation. We recently brought our findings and ideas together to develop a novel treatment strategy for tonal tinnitus - tailor-made notched music training (TMNMT) [3-6]. By notching the music energy spectrum around the individual tinnitus frequency, we are attracting lateral inhibition to auditory neurons involved in tinnitus perception.

The contemporary view on tinnitus biology is that although tinnitus may be triggered by injury to the inner ear, the neural generators are most readily found centrally, and while the neural generators may be primarily auditory, non-auditory centers often participate. Studies of noise-induced tinnitus have given rise to the general theory that tinnitus is triggered by injury to inner ear hair cell populations. The consequence is a lack of lateral inhibition from the primarily damaged frequency areas, which leads to augmented excitation in the spectral neighbor’s regions of the lesion. This change causes plastic adjustments in the central auditory system that culminates in alterations of spontaneous activity. The theory also holds that central auditory system plasticity is the main centerpiece of these adjustments, whereby reduced auditory nerve input triggers a shift in the balance of excitation and inhibition centrally [7]. This shift leads to the emergence of a tripartite complex of changes that includes hyperactivity, increased bursting activity, and increased synchrony. Such changes reflect a loss of inhibitory drive to neurons, particularly of glycinergic and GABAergic systems, but increases in excitation via upregulations of glutamatergic and cholinergic systems [8-11]. This view is consistent with the three-faceted nature of tinnitus, which includes auditory, attentional, and emotional components.

Based on the above theory, tinnitus is a result of hyper-activity/hyper-synchrony of auditory neurons coding the tinnitus frequency, which has developed to synchronous mass activity owing the lack of inhibition. It can be assumed that removal of exactly these frequency components from an auditory stimulus will cause the brain to reorganize around tonotopic regions coding the tinnitus frequency. Based on this assumption, a novel treatment for tonal tinnitus has been introduced and evaluated in two controlled blind studies [3,6]. The auditory stimulus was the favorite music as chosen by the individual patient in order to increase the positive attention. However, the music was filtered to exclude a range around the individual’s tinnitus frequency. To monitor the effectiveness of this treatment, a behavioral self-report measure was used in addition to two neuro-physiological measures of activity of the auditory cortex: the Auditory Steady State Response (ASSR), representing primary auditory cortex activation, and the N1m response, representing activity of secondary auditory cortical areas. After 6 and 12 months of TMNMT, clear effects of the therapy have been obtained. The tinnitus frequency showed a significant decrease in both the behavioral and the physiological responses compared to controls. The subsequent short-term (5 days) training study [6] indicated that training was more effective in the case of tinnitus frequencies ≤ 8 kHz compared to tinnitus frequencies > 8 kHz, and that training should be performed over a long-term period in order to induce more persistent effects. This phenomenon could be explained by attraction of lateral inhibition to the neurons coding the tinnitus frequency, which they obtain from those neurons coding frequencies above and below the tinnitus frequency. This mechanism explains the alleviation of the tinnitus perception and thus the improvement reported by the tinnitus patients.

Based on the findings of and ideas triggered by those studies, we further developed both the training stimulus and the training strategy in order to maximize their efficacy. Thus, this refined, completely non-invasive, and relatively low-cost treatment approach to tonal tinnitus will be evaluated in a randomized controlled clinical trial on a larger cohort of tinnitus patients.

## Methods/design

### Trial design

This will be a Randomized Control Trial (RCT) in parallel group design. RCTs are the gold standard for evaluating the efficacy of a treatment intervention [12] and our RCT will be performed in a double-blinded manner in order to control for non-specific and placebo effects. The choice of the intervention we are going to apply is based on “proof of concept” studies in humans [3-6] and on a recent animal study [13].

We will investigate a target and a placebo group. Using a placebo treatment as a control condition serves to differentiate specific effects of an intervention from non- specific effects like expectation, anticipation, patient care, the investigator's attention, as well as plain listening to music and the accompanied relaxation or spontaneous improvement. The treatment will last 3 months, with two successive hours of TMNMT per day (ca. 180 hours of training in total). The target and placebo groups will receive the corresponding treatments as described below.

**Figure 1:**
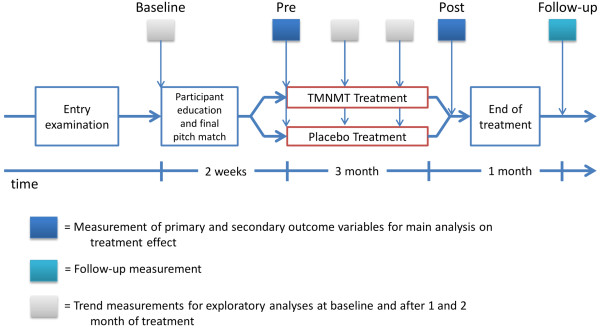
Tinnitus clinical trial design.

The clinical trial design is graphically presented in Figure 1. Participants are recruited in an entry examination. The procedure is described below. On the second date (baseline) participants are informed about the clinical trial and are instructed how to perform a pitch matching of their tinnitus during the following week. On the third (pre) date, two weeks later, participants are finally selected for the trial. The decision is based on a reliable performance on the pitch-matching task. Participants are then provided with the music treatment. Primary and secondary outcome measures are collected at baseline, initially before treatment starts (pre), at the end of the treatment (post) and at one-month follow-up. Trend measurements consist of the primary outcome measures.

### Participants (inclusion and exclusion criteria)

The participants (estimated N = 100) are recruited by means of advertisements in local newspapers, our homepage, the homepage of the Faculty of Medicine of the University of Münster and by the distribution of flyers to local ENT practitioners. We overtly communicate the study criteria through homepages and flyers. Additionally, potential participants are asked to answer a questionnaire covering questions like the characteristics, duration, onset, possible cause, and treatment of the tinnitus as well as pre-existing illnesses, hyperacusis, current medication and drug consumption. According to the questionnaire, the participants are scheduled for a multidisciplinary entry investigation in the ENT department of the University Hospital Münster. This procedure lasts for approximately two hours. It starts with a screening by psychologists who explore in depth the occurrence, the development, and the treatment of the tinnitus as well as the criteria already specified in the questionnaire described above. Furthermore, tinnitus is assessed by means of standardized questionnaires (Tinnitus Handicap Questionnaire (THQ) [14], Tinnitus Handicap Inventory (THI) ( [15] German translation), and VAS). Psychological assessment is additionally supported by psychometrical data of a depression scale (Allgemeine Depressionsscala – Langfassung, ADS-L) [16] and the Symptom Checklist to capture general psychological distress (SCL-90-R) [17]. After psychological assessment, a physical examination of the ear is performed by an ENT physician. Additionally, hearing thresholds of the tinnitus patients are measured via an audiometric procedureusing a clinical audiometer (Type Madsen Astera, Denmark) that is able to operate in an extended frequency range up to 16 kHz.

The patients recruited for the clinical trial will meet all inclusion criteria. In case of bilateral tinnitus, the dominant tinnitus frequency should not differ between ears according to patients’ reports.

#### Inclusion criteria

1. (i) Patients with chronic (≥ 3 months) tonal (i.e. peep- or whistle-like) tinnitus with (ii) dominant tinnitus frequencies between 1 and 12 kHz, and (iii) without severe hearing loss (≤ 70 dB HL) in the frequency ranges of one half octave above and below the tinnitus frequency.

2. Aged between 18 and 70 years.

3. Written informed consent to participate in the trial.

#### Exclusion criteria

1. Report of severe or acute neurological or psychiatric disorders (e.g. amnesia, dementia, epilepsy, etc.) that could limit their capability or that could result in major difficulties regarding the patients’ motivational compliance, their ability to follow instructions or to participate in the training.

2. Tinnitus patients with acute otological diseases.

3. Planned start of other rehabilitation therapies that might interfere with this trial, participation in another clinical trial, or insufficient motivation to participate.

#### Drop-out criteria

Participants are declared as “drop-outs” if they indicate that they do not want to continue the TMNMT. If patients experience increase of tinnitus loudness or state to hear an additional tone after they listened to the music for at least eight weeks, we advise them to stop their participation in the training. Nevertheless, we ask participants who stop performing the training to continue completing questionnaires.

However, we are aware that a well-characterized study sample increases the chances to find an effective intervention, but the generalizability of the results is reduced since the study population is no longer representative of the majority of tinnitus patients [18].

### Music spectrum treatment

**Figure 2:**
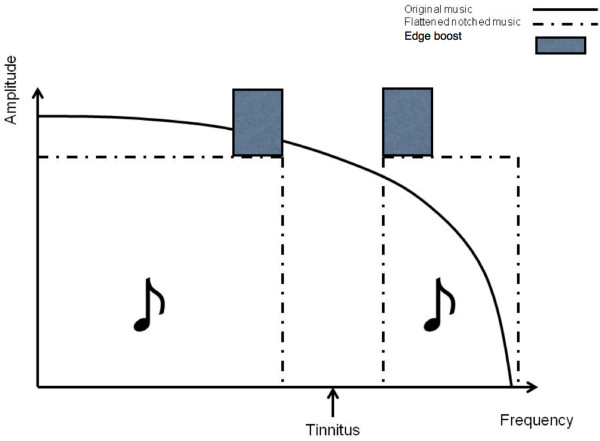
Music spectrum modification.

The patients from target and placebo treatment groups provide their favorite music (10 CDs). The music will then be modified “on-line” in several successive steps. First, the energy spectrum of the music will be “flattened”, i.e. the amplitude of the music frequency spectrum in the low and high frequency range will be equalized by redistribution of energy from low to high frequency ranges. This should guarantee an equal amount of spectral power below and above the frequency area suppressed by the notch filter. Second, the fre-quency band of 1/2 octave width centered at the individual tinnitus frequency will be removed from the music energy spectrum. Third, the edges of the notch will be amplified within the width of 3/8 octaves on each side of the notch by 20 dB SL [19]. All three procedures are aimed at increasing the lateral inhibition effect within the notch area corresponding to the tinnitus frequency (c.f. Figure 2). The performance of the individual TMNMT procedure as described in our “proof of principle” papers will be performed according to one of two protocols: (i) target (fixed notch) or (ii) placebo (moving notch) and is realized on a mobile device, which provides a tremendous advantage allowing to perform the TMNMT at home.

#### Target condition

In the target condition, the frequency band centered at the individual tinnitus frequency of each tinnitus patient is removed from the music energy spectrum, and the music is modified due to changes described above. The music is delivered to both ears simultaneously and modified identically. The patients listen to their individually modified treatment music daily in a quiet environment via supplied closed headphones (model: Sennheiser HD 201) with convenient loudness over the course of 3 months. Patients are told not to listen, if possible, to normal, non-modified music during the treatment phase. Listening times and duration are documented on a daily basis by a specially developed iOS application. The obtained data are sent to the tinnitus research team as an email attachment every week.

#### Control (Placebo) condition

In the placebo condition, a moving notch filter having the same bandwidth as in the target condition but omitting the tinnitus frequency region is applied. The moving filter randomly chooses a frequency band outside of the tinnitus frequency region. After 5 s of filtering, the center frequency of the filter randomly jumps either 1/18 octave up or down and continues jumping in the same direction every 5 s until its lower or higher edge reaches a predefined border, at which point it changes direction.

#### Assessment of the tinnitus frequency

We adapted a recursive two-interval forced choice estimation of the tinnitus frequency [20,21], which is as good as the clinical audiometry testing, but it can be performed in one third of the time [22]. The frequency range (1 to 16 kHz) is bisected into two equally large subintervals. For both, the low and the high frequency subinterval, the participant has to choose whether the tinnitus is more similar to its lower or higher frequency end.

Depending on the outcome, bisection and two-interval forced-choice testing are reapplied to the low subinterval, the high subinterval, or a new middle interval that is bound by the midpoints of the low and the high subinterval [20,21]. Prior to testing, the left or right ear is selected. Loudness of the test tones is adjusted individually by participants to match the tinnitus loudness and saved by the application. A two-alternative forced-choice octave confusion test completes the testing. All choices made by participants are saved in an ASCII table file. Participants perform the pitch-matching test at home two times a day over five days. Frequencies are transformed into Cent. The Cent is a logarithmic unit of measure used for musical intervals. Twelve-tone equal temperament divides the octave into 12 semitones of 100 cent each. With the frequency in Cent the arithmetic mean of the ten measurements is calculated as the final tinnitus frequency for the treatment.

#### Software for music modification

To perform the necessary modification of the music (TMNMT), software which performs the three successive steps (described above) has been developed. In the target condition, the investigator has to set up a center frequency and a bandwidth of the notch filter and choose a bandwidth for the amplified notch edges. These parameters are entered via a graphical user interface provided by the software application. In the placebo condition, the settings are defined using the same graphical user interface.

#### Instructions for subjects during TMNMT

Subjects are instructed to listen to the music attentively in a quiet surrounding at home with comfortable loudness. They are allowed to read, surf the internet or do other relaxing activities. Any cognitive or attention demanding task, such as working or learning, should be avoided. Additionally, there should be no sound distractions during TMNMT, such as telephone or alarm bell ringing, conversations etc.

### Outcome measures

Since tinnitus is a purely subjective percept, the definition of outcome measurements is challenging [18]. Comprehensive assessments of tinnitus would ideally address four subjective principal components: (1) auditory features of the tinnitus percept including loudness and pitch, (2) emotional distress, (3) attentional features like awareness of tinnitus in daily life and (4) behavioral influence. Separate assessment of these different components is especially relevant, since they correlate only relatively weakly with each other [23]. The primary efficacy endpoints are significant reductions in tinnitus loudness, annoyance, awareness and handicapping scores at the end of the training after 3 months compared to baseline. These parameters are monitored throughout the study, i.e. prior to TMNMT start (baseline), during TMNMT (treatment), after TMNMT (post) and one month after TMNMT (follow-up) (c.f. Figure 1).

#### Primary outcome measures

(i) Tinnitus-related distress is assessed with the THQ ( [7] German translation). For statistical analyses, the total score will be used.

(ii) VAS total score. Participants will rate their tinnitus loudness, annoyance, awareness and handicap by VAS (scale from 0 to 100). The mean score from these four scales will be used as the total score.

### Secondary outcome measures

Following the suggestion of Landgrebe et al. [18] we include the THI ( [15] German translation) for better comparability among clinical trials in tinnitus. Additionally, the German version of the Tinnitus Questionnaire (TQ) [24,25] will be used as secondary outcome measures. Furthermore, the emotional and cognitive distress subscale of the THQ [14] and the subscales of the VAS will be analyzed.

To allow further sub-group analyses, we will collect the following data: ADS-L, SCL-R-90, German hyperacusis questionnaire (Geräuschüberempfindlich-keits-Fragebogen, GÜF), listening duration of TMNM, average listening duration of conventional music, VAS measuring different aspects of listening to TMNM (enjoyment, relaxation, attention to the music), subjective expe-rience with TMNMT (improvement of tinnitus, recommendation of TMNMT) and side effects of TMNMT.

To evaluate the time course of subjective tinnitus changes we will measure the subjective tinnitus loudness, annoyance, distress, and handicap status by means of VAS throughout the study on two days per week (Wednesday and Sunday) beginning fourteen days prior to TMNMT onset and ending 31 days after TMNMT completion. The participant is reminded to do those ratings via the calendar function of the provided mobile device twice a week, every Wednesday and Sunday at 12:00 o’ clock.

### Sample size

During the planning state, a priori power calculations were used to estimate an adequate sample size to find meaningful clinical changes in our outcome variables. The calculations were performed using G*Power3.1.5 [26]. The estimation of the expected effect size was based on an approximation rule of thumb about the minimal clinically important differences proposed by Norman et al. 2003 [27]. In their systematic review of 38 studies, they found that the threshold to determine a clinical relevant change in health-related questionnaires was approximately 0.5 standard deviations [27]. This corresponds to a standardized effect size of d = 0.5 which can be interpreted as a medium effect [26]. The conventions for effect sizes of repeated measures ANOVAs were defined by Cohen [28] as f = 0.1, f = 0.25 and f = 0.4 for small, medium and large effects, respectively. Thus, we chose a medium effect size of f = 0.25 for our power analysis to assure that we are able to detect the assumed minimal clinically important difference. We planned to achieve a power of .90 for testing the interaction effect Time (pre, post) × Group (placebo, treatment) (5% significance level). The correlation between the two repeated measures was set to r = 0. Note that this reflects a conservative approximation and any correlation larger than r = 0 would reduce the required amount of participants. Due to these assumptions, a sample size of N = 88 resulted as necessary. To take into account an anticipated dropout rate of 13%, the final sample size will be set to N = 100, i.e. 50 participants per group.

### Allocation, randomization and blinding

As of now, we are planning to investigate 100 patients with tonal tinnitus (see recruitment criteria above) in two study arms:

(iii) Target group, 50 tonal tinnitus patients with tinnitus frequency (TF) < = 12 kHz obtaining TMNMT

(iv) Control group, 50 tonal tinnitus patients (TF < = 12 kHz) obtaining placebo treatment.

The allocation to the two groups will be done using stratified randomization. Stratification is an appropriate allocation method for improving power in small trials (< 400 patients) and preventing type 1 errors [29]. It was proposed to use a minimum number of strata to improve statistical efficiency, since this allows the assignment of an equal number of subjects to each treatment and assures an equal distribution of the stratification factor between groups [29,30]. We choose the variables age (< 51 or > = 51 years) and hearing loss (< 40 dB or > = 40 dB) [31,32] as stratification categories to keep the number of strata minimal (n = 4) but include the two variables most probable to influence the treatment outcome. Within each stratum, a blocked randomization will be performed using a computer generated randomization list. To ensure that study results are not confounded by anticipation or expectation, both the participants and the investigators will be blind to the allocation.

Whether the participant receives placebo or real treatment is determined by the settings of the TMNMT-App on the iPod touch mobile device. An external person, not involved in the Clinical Trial, is responsible for the allocation of each participant to one of the two groups (treatment or placebo) and sets up the App according to that. Afterwards, the App is locked and password-secured to disable further changes and the iPod is handed back to the original investigator.

### Statistical methods

The statistical analysis for this clinical trial will be performed at the Institute for Biomagnetism and Biosignalanalysis. The respective hypotheses are formulated as one-sided [3-6]. In an intention-to-treat analysis the data of all participants that were assigned to the treatment or to the placebo group will be analyzed statistically. In anticipation of dropouts and missing data, a likelihood-based mixed-effects model for repeated measures analysis [33] was chosen as the primary analysis.

Additionally, we will run an analysis with complete cases. This will be done by means of repeated measures ANOVAs and planned comparisons; post-hoc analysis will be corrected for multiple testing, if necessary. In order to rule out adverse effects of the treatment and to prevent futility, an interim analysis will be conducted after 50% of the participants completed the training (post measurement). For this purpose data will be unblinded and stored in a way that no inference to the single participant can be made (e.g. without age, gender, hearing loss, etc., only analysis relevant data (group and outcome measures)). The storage is done by an external person, who is also responsible for blinding, and the analysis is carried out by the investigators.

In case of a significant interaction between group and primary outcome measures revealing a negative effect for the treatment group, the trial will be stopped.

### Reporting of trial results

The last and very important step in planning and conducting a clinical trial is to report the results (even if negative). We further agree that publishing results from clinical trials is essential in order to inform the scientific community of what was done and what the results are. Therefore, it is necessary that negative results should also be published.

Furthermore, it is opinion that the results of the clinical trial should be published following a common scientific standard set by the CONSORT Group (“consolidated standards of reporting clinical trials” http://www.consortstatement.org) [34,35]. The main objective of CONSORT is to facilitate critical appraisal and interpretation of RCTs by providing guidelines on how to report methods and results of a RCT. We are informed that the methodological details, which are required to be reported, are summarized in the CONSORT statement and checklist (http://www.consort-statement.org/consort-statement).

### Ethical aspects of the clinical trial

The ethical aspects of the clinical trial were reviewed by an independent Institutional Review Board (Ethical Commission of the University of Münster) and an ethical approval was obtained before the trial starts. In addition, according to Good Clinical Practice-(GCP)-Guidelines, the participants in the clinical trial are fully informed about the nature, benefits and potential dangers of the trial as well as alternative treatment options, and they have to sign an informed consent form before they can be enrolled into the study (c.f. the information and consensus forms). If the target treatment is effective and superior to placebo, the patients of the placebo group will be offered the target treatment after completion of the trial.

## Discussion

This is the first randomized controlled trial on a larger number of patients with tonal tinnitus applying the TMNMT. Our data will verify more securely and reliably the effectiveness of this kind of completely non-invasive and low-cost treatment approach on tonal tinnitus.

## Abbreviations

TMNMT: Tailor-made notched music training; RCT: Randomized control trial; MEG: Magnetoencephalography; ASSR: Auditory steady state response; THQ: Tinnitus handicap questionnaire; VAS: Visual analogue scales; THI: Tinnitus handicap inventory; TQ: Tinnitus questionnaire; TF: Tinnitus frequency; ADS-L: Allgemeine depressions skala-langfassung; SCL-90 R: Symptom checkliste.

## Competing interests

The authors declare that they have no competing interests.

## Authors’ contributions

CP and CR developed the concept of the trial and applied for funding. CP, AS, RW, AE, PL and AW contributed to the development and to the design of the protocol, developed the analysis plan, worked out the technical and technological preparations for the trial, developed the logistic for recruitment of the patients with tonal tinnitus and will perform the trial. CP has drafted the manuscript with critical input from all other authors who have read and approved the final manuscript.

## Authors’ information

CP: University Professor, Dr.; Director of the Institute for Biomagnetism and Biosignalanalysis, University of Münster

CR: University Professor, Dr.; Director of the ENT Department, University Clinic Münster, University of Münster

AS: Dipl. Psych., PhD candidate at the Institute for Biomagnetism and Biosignal-analysis, University of Münster

RW: Dipl. Psych., PhD candidate at the Institute for Biomagnetism and Biosignal-analysis, University of Münster

AE: Dipl. Psych., PhD candidate at the Institute for Biomagnetism and Biosignal-analysis, University of Münster

PL: Dipl. Psych., PhD candidate at the Institute for Biomagnetism and Biosignal-analysis, University of Münster

AW: Dipl. Ing. Institute for Biomagnetism and Biosignalanalysis, University of Münster

AS: MS Psych., PhD candidate, 2/22 Foyle Road, Bayswater, WA 6053, Australia

## Pre-publication history

The pre-publication history for this paper can be accessed here:

http://www.biomedcentral.com/1471-2377/14/40/prepub
